# Colonization of Wheat, Maize and Cucumber by *Paenibacillus polymyxa* WLY78

**DOI:** 10.1371/journal.pone.0169980

**Published:** 2017-01-11

**Authors:** Tianyi Hao, Sanfeng Chen

**Affiliations:** State Key Laboratory of Agrobiotechnology and College of Biological Sciences, China Agricultural University, Beijing, P. R. China; Agroecological Institute, CHINA

## Abstract

*Paenibacillus polymyxa* WLY78 is a nitrogen fixer and it can be potentially applied to biofertilizer in agriculture. In this study, *P*. *polymyxa* WLY78 is labelled with gfp gene. The GFP-labelled *P*. *polymyxa* WLY78 is used to inoculate wheat, maize and cucumber seedlings grown in the gnotobiotic system and in soil, respectively. Observation by confocal laser scanning microscope reveals that the GFP-labeled bacterial cells are mainly located on the root surface and epidermis of wheat, and only a few cells are present within cortical cells. In maize and cucumber seedlings, bacterial cells were colonized in epidermal and cortical cells, intercellular spaces and vascular system of root, stem and leaf tissue interiors besides on root surfaces. Higher densities of the bacterial cells in roots, stems and leaves indicated that *P*. *polymyxa* WLY78 cells could migrate from roots to stems and leaves of maize and cucumber. This study will provide insight into interaction between *P*. *polymyxa* WLY78 and host cells.

## Introduction

There is a close correspondence between fertilizer consumption and cereal crop and vegetable production. Thus, the use of chemical nitrogen fertilizers (mainly NH_3_, NO_3_^−^and urea) has been a key aspect in increasing cereal crop and vegetable productivity [1]. However, excessive application of nitrogen fertilizers on cereal crops and vegetables in China has led to soil compaction and environmental pollution [2].

Biological nitrogen fixation—-the conversion of atmospheric nitrogen into ammonia by symbiotic, associative and free-living bacteria—is an alternative to the use of chemical nitrogen fertilizers. Inoculation of non-legume crop plants with different N_2_-fixing inoculants has proved useful in the reduction of chemical nitrogen fertilizer [3, 4].

*Paenibacillus polymyxa* WLY78, isolated by our laboratory from the rhizosphere of bamboo, is capable of fixing nitrogen and producing antimicrobial substances [5, 6]. Just as some *Paenibacillus* strains, e.g. *P*. *beijingensis*1-18, *P*. *polymyxa* 1–43, *Paenibacillus* sp. 1–49, and *Paenibacillus* sp.1-33 increased 5.1~26.9% wheat yields by inoculation [7], *P*. *polymyxa* WLY78 also could increase about 10% wheat and maize yields (results not published). However, whether *P*. *polymyxa* WLY78 can colonize in these plants is not yet known. In this study, *P*. *polymyxa* WLY78 was labeled with GFP and then the GFP-labeled bacterium was used to inoculate wheat, maize and cucumber roots. The colonization patterns of *P*. *polymyxa* WLY78 in these plants were observed under the confocal laser scanning microscope. Our study will not only reveal the colonization patterns on different plants, but also provide clues for studying the plant-growth promoting traits in *P*. *polymyxa*.

## Materials and Methods

### Bacterial strain and culture conditions

*P*. *polymyxa* WLY78, which was isolated by our laboratory from roots of bamboo, was used here [5]. The bacterium was cultured at 30°C in Luria-Bertani (LB) medium.

### Construction of plasmid and GFP-labelled *P*. *polymyxa* WLY78

In order to obtain GFP-labelled *P*. *polymyxa* WLY78, a plasmid vector which carried a *gfp* gene and could replicate in *P*. *polymyxa* was here constructed. The pHY300PLK plasmid, which was a shuttle vector in both *E*. *coli* and *B*. *subtilis*, was used as a vector backbone [8]. A *gfp* (mut3a) gene together with its promoter in a 4.4-kb DNA fragment was obtained from plasmid pGFP4412 by digestion with *Eco*RI and *Hin*dlII [9]. Then the 4.4-kb DNA fragment was ligated to the shuttle vector pHY300PLK digested with *Hin*dIII and *Eco*RI restriction enzymes, thus yielding the plasmid pGFP300 which carries a *gfp* (mut3a) gene. The competent cells of *P*. *polymyxa* WLY78 were prepared as described [10]. GFP-labeled *P*. *polymyxa* WLY78 was obtained by transferring pGFP300 into competent *P*. *polymyxa* WLY78. The GFP-labelled *P*. *polymyxa* WLY78 was observed under Olympus FluoView™ FV1000 confocal microscope.

### Plant growth and inoculation

Wheat seeds were surface-sterilized by soaking in 20% sodium hypochlorite solution for 20 minutes, and then they were rinsed repeatedly with sterile water, and finally the seeds were soaked in sterile-distilled water for 4 hours. Then the seeds were placed on the wet filter papers in sterile dishes at room temperature, and after about 10 days later, the root seedlings were approximately 2 centimeter (cm) in length. Similarly, maize and cucumber seeds were surface-sterilized and then germinated.

For the gnotobiotic system, the germinated seedling was transferred to a sterile flask (6 cm in diameter and 10 cm in height) filled with 100 mL 1/2 × Murashige & Skoog (MS) semisolid agar medium [11]. Then 20 mL of the GFP-labelled *P*. *polymyxa* WLY78 cells (2.4×10^8^ cells/ml) were inoculated to the flask.

For soil system, the germinated seedling was transferred to a flowerpot. Each flowerpot was filled with sterilized soil and the concentration of the GFP-labelled bacterial cells in soil was about 1.74×10^7^ cells/g soil. The plants were grown in a light growth chamber at 27℃ with 16 hours of light per day and 70% humidity.

### Laser confocal microscopic observation

In the gnotobiotic system, the plants were taken out from the semisolid agar medium after inoculation for certain times. Plant root surfaces were rinsed with sterile water and then were directly observed under the laser confocal (Olympus FluoView™ FV1000 confocal microscope or Zeiss LSM 710 confocal microscope). Some tissues of roots, stems and leaves were sectioned in longitudinal and transverse directions to examine the colonization of GFP-labelled *P*. *polymyxa* WLY78. In the soil system, the plants were taken out from the soil, rinsed with sterile water and observed directly under the laser confocal. The resulting images were obtained using the FV10-ASW 4.0 Viewer software or Zen 2012 software.

## Results

### Construction of GFP-labeled *P*. *polymyxa* WLY78

*P*. *polymyxa* WLY78 is a nitrogen-fixing bacterium with great potential use as biofertilizer in agriculture. In order to investigate the interaction between the bacterial cells and plants, *P*. *polymyxa* WLY78 was labelled with the green fluorescence protein (*gfp*) gene. As was shown in Materials and Methods, GFP-carrying plasmid pGFP300 which is able to replicate in both *B*. *subtilis* and *E*. *coli* is here constructed and transformed into *P*. *polymyxa* WLY78. Three positive transformants with tetracycline resistance (Tc^R^) are obtained. Colony PCR analysis showed that each of the three transformants carries the *gfp* gene.

**Fig 1:**
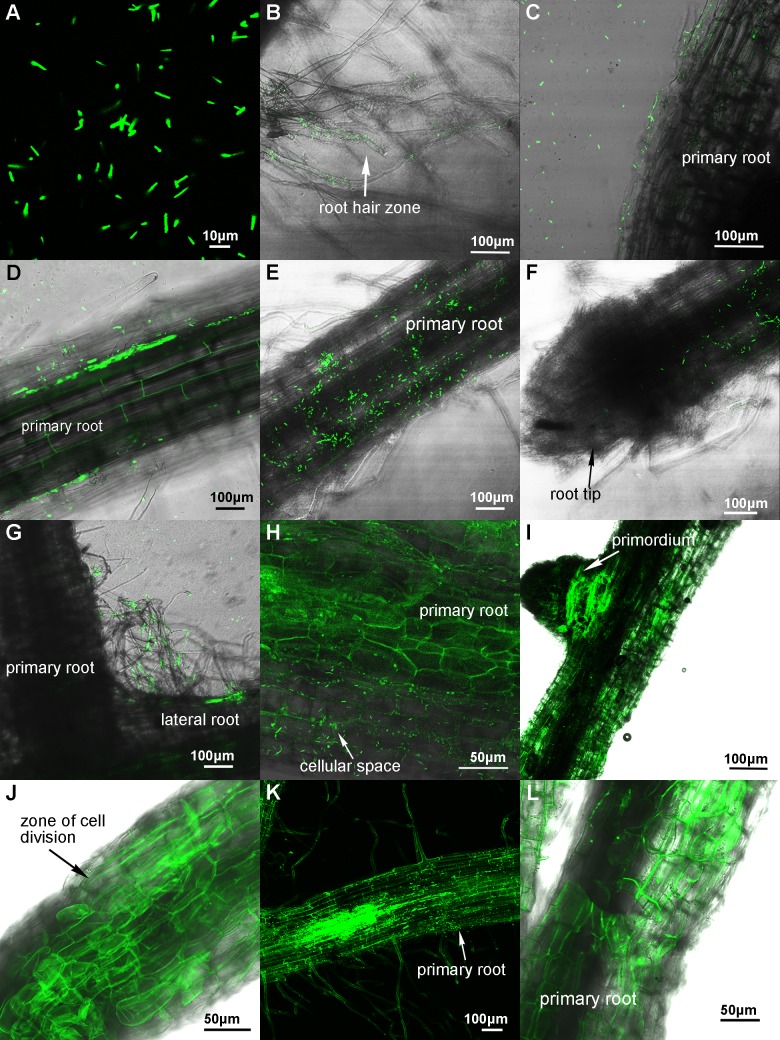
Confocal image of the GFP-labeled *P*. *polymyxa* and colonization of the GFP-labeled cells in wheat roots in the gnotobiotic system. (A) Confocal image of the GFP-labeled *P*. *polymyxa* cells. (B-D) Colonization patterns of the GFP-labeled *P*. *polymyxa* WLY78 in wheat roots after 15 hours of inoculation. (E-G) Colonization patterns after 1 day of inoculation. (H) Colonization patterns after 3 days of inoculation. (I-J) Colonization patterns after 5 days of inoculation. (K) Colonization patterns after 7 days of inoculation. (L) Colonization patterns after 10 days of inoculation.

The confocal laser scanning microscopy (CLSM) observation shows that the GFP-labeled *P*. *polymyxa* cells emit bright green fluorescence (Fig 1A). Furthermore, we found that the GFP-labeled *P*. *polymyxa* cells were moving actively under microscopy.

The stability of GFP-carrying plasmid in *P*. *polymyxa* WLY78 is very important for monitoring colonization of plants. In order to assess this property of the GFP-labeled *P*. *polymyxa* WLY78, the bacterial cells were cultivated in LB with tetracycline (25 μg/μL) to the phase of logarithmic growth, and then they were transferred to the fresh LB medium in the absence of antibiotic pressure with the dilution of 1:100. After three days of incubation in LB medium, the GFP-labeled *P*. *polymyxa* WLY78 cells were still able to emit green fluorescence, suggesting that *gfp* gene was stably expressed in *P*. *polymyxa* WLY78.

### Colonization of wheat roots by GFP-labeled *P*. *polymyxa* WLY78 in the gnotobiotic system

The GFP-labelled *P*. *polymyxa* WLY78 was used to inoculate wheat seedlings grown in the gnotobiotic system and in soil, respectively. The gnotobiotic model system can be used for studies on the interaction between *P*. *polymyxa* and plants, while soil system might reflect the natural pattern of colonization in soil system.

The wheat roots at 15 hours and 1, 3, 5, 7, 9, and 11 days after inoculation, respectively, were cut or sectioned from the plants, and then the root sections were examined under a laser confocal microscope. 15 hours after inoculation, the GFP-labelled *P*. *polymyxa* cells were found to colonize in the root hair zone and on the surface of the primary roots (Fig 1B–1D). One day after inoculation, a significant proportion of the bacterial population irregularly scattered on the primary root surface (Fig 1E). However, there were no bacterial cells in the region of the root tip (Fig 1F). At the same time, the bacterial cells were also found in the junction of the primary and lateral roots (Fig 1G). The bacterial cells appeared to be more abundant in the cellular space of epidermal cells after 3 days of inoculation (Fig 1H).

As shown in Fig 1I and 1J, five days after inoculation, the stronger fluorescence on the root surface indicates that bacterial population densities were increased, and the bacterial cells were abundant in the region of the lateral root primordium (Fig 1I) and the zone of cell division (Fig 1J). Z axis-optical section, which is a quasi-three dimensional technique, is here used to detect the depth of bacterial infection at seven days after inoculation and it was found that a fluorescence spot became about 30 μm in width and 60 μm in length (Fig 1K), indicating that the GFP-labeled *P*. *polymyxa* cells expand within the wheat roots. Ten days after inoculation, it was found that *P*. *polymyxa* cells were still tightly colonized on the surface of roots (Fig 1L).

### Colonization of *P*. *polymyxa* WLY78 in wheat roots in the soil system

In this study, inoculation of the GFP-labeled *P*. *polymyxa* WLY78 to wheat planted in soil was also performed. This system is more similar to the natural environment. The root samples were flushed with water before observation under laser confocal microscope.

**Fig 2:**
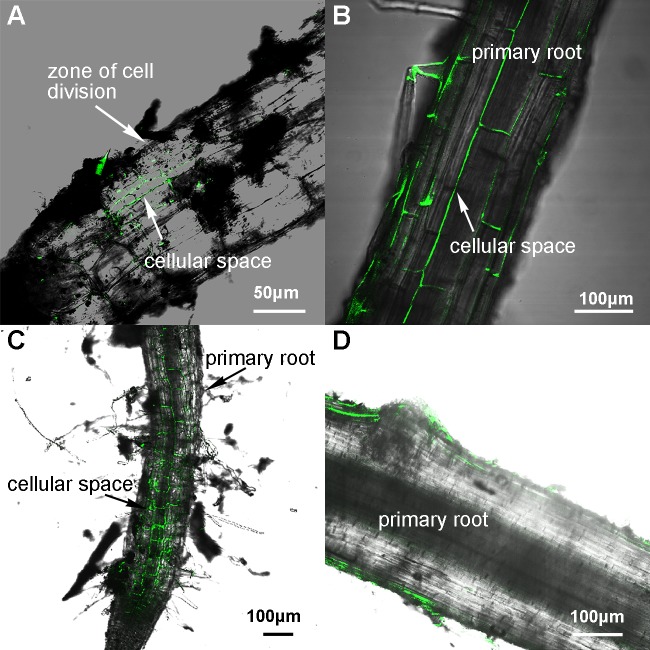
Colonization of the GFP-labeled *P. polymyxa* WLY78 in wheat roots in the soil system. **(A)** Colonization patterns after 3 days of inoculation. (B) Colonization patterns after 7 days of inoculation. (C-D) Colonization patterns after 10 days of inoculation.

As shown in Fig 2A, after three days of inoculation, bacterial cells attaching on the surface of roots could be observed. Seven days after inoculation, the bacteria were found in a straight line along inter cellular space of epidermal cells (Fig 2B). Ten days after inoculation, bacterial cells extended to the whole wheat roots (Fig 2C) and the longitudinal sections of roots showed that the majority of the bacterial cells were distributed on the root surface (Fig 2D).

Compared to that in gnotobiotic system, bacterial population on the roots in the soil system is much lower. We think that the lower density of bacterial cells on the roots in the soil system may be caused by washing roots before microscopy observation.

### Colonization of maize by GFP-labeled *P*. *polymyxa* WLY78 in the gnotobiotic system

**Fig 3:**
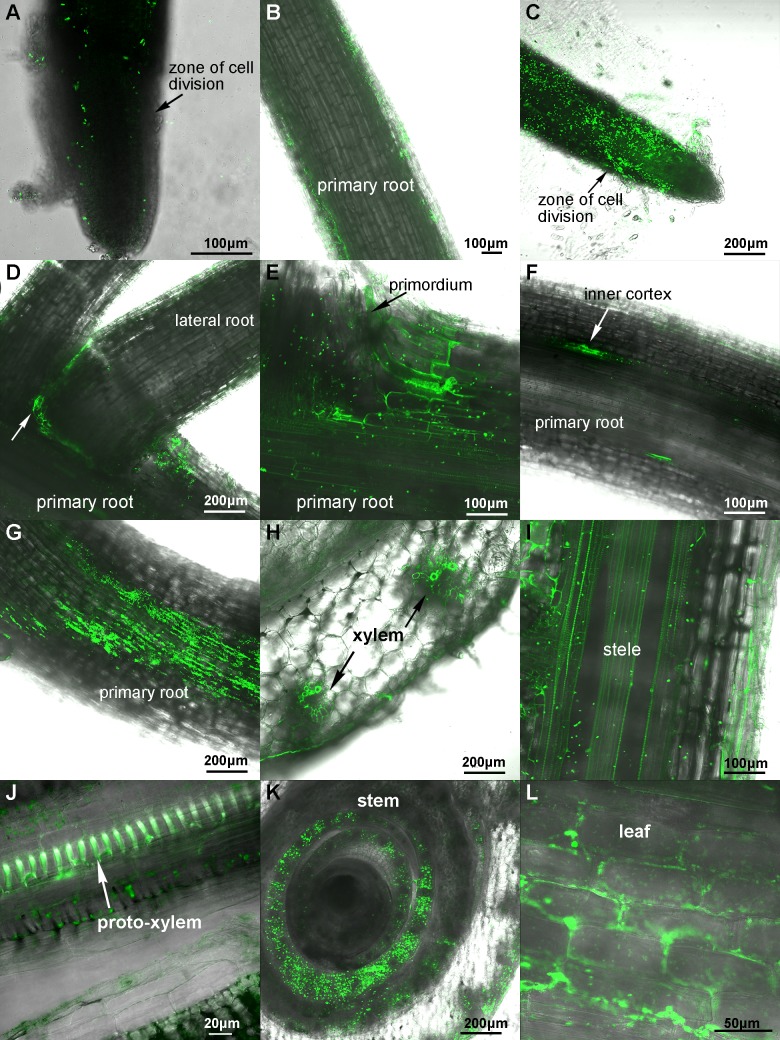
Colonization of the GFP-labeled *P*. *polymyxa* WLY78 in maize seedlings in the gnotobiotic system. (A-B) Colonization patterns after 1 day of inoculation. (C-D) Colonization patterns after 3 days of inoculation. (E-F) Colonization patterns after 5 days of inoculation. (G-H) Colonization patterns after 7 days of inoculation. (I-L) Colonization patterns after 10 days of inoculation.

Similarly, the GFP-labelled *P*. *polymyxa* WLY78 was used to inoculate maize seedlings grown in the gnotobiotic system. One day after inoculation, GFP-labeled bacterial cells were found to be attached to the surface of maize roots (Fig 3A and 3B). Three days after inoculation, a higher density of bacterial cells were found to be attached to the root surface, especially in the meristematic zone (Fig 3C) and the junctions of the primary and lateral roots (Fig 3D). Five days after inoculation, the bacteria cells were found within epidermis cells and the space of epidermis cells of maize roots (Fig 3E). Longitudinal sections of primary roots showed that bacterial cells were distributed within cortex and vascular bundle (Fig 3F). After one week of inoculation, A significant colonization was found by bacterial cells on the root surface (Fig 3G). Moreover, transverse sections showed that the bacterial cells were in vascular bundle of stem (Fig 3H). Ten days after inoculation, longitudinal sections showed that cortex and stele of both roots (Fig 3I) and stems (Fig 3J) were already heavily colonized by bacterial cells. Transverse sections also showed that bacterial cells were distributed inside the vascular bundles of stem (Fig 3K). And a large number of bacterial cells were also found in leaves (Fig 3L).

### Colonization of cucumber by GFP-labeled *P*. *polymyxa* WLY78 in the gnotobiotic system

**Fig 4:**
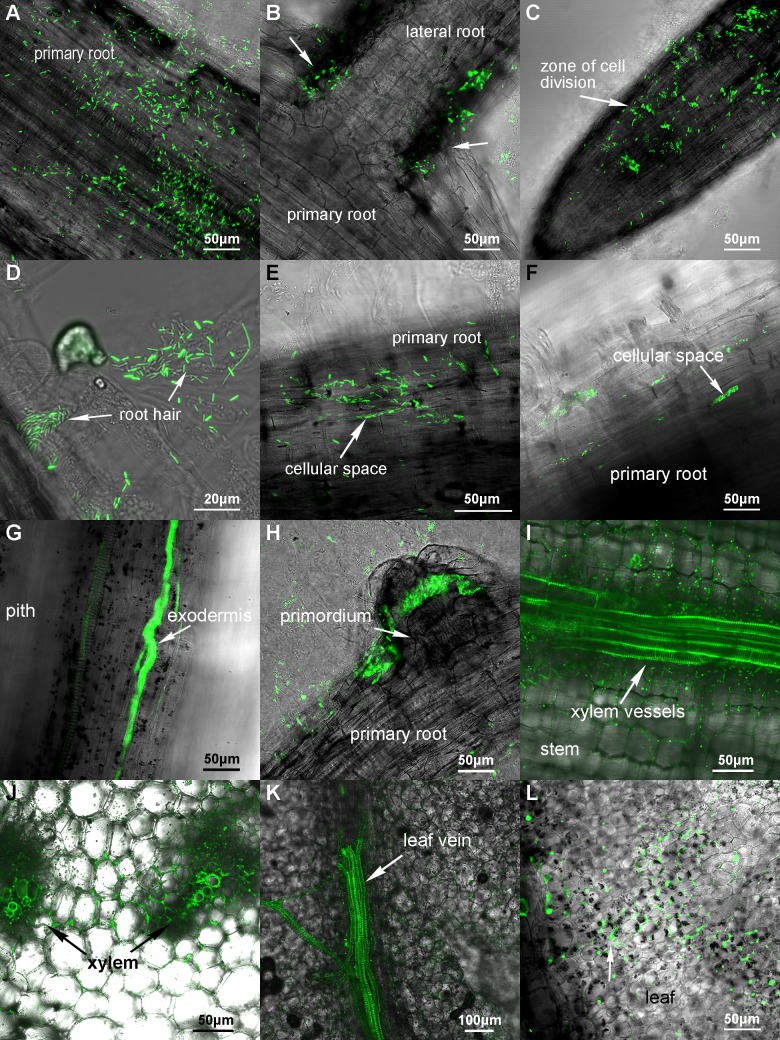
Colonization of the GFP-labeled *P*. *polymyxa* WLY78 in cucumber seedlings in the gnotobiotic system. (A-D) Colonization patterns after 1 day of inoculation. (E-H) Colonization patterns after 3–5 days of inoculation. (I-J) Colonization patterns of cucumber stems after 7 days of inoculation. (K-L) Colonization patterns of leaves after 10 days of inoculation.

The GFP-labelled *P*. *polymyxa* WLY78 was also used to inoculate cucumber seedlings grown in the gnotobiotic system. One day after inoculation, a significant proportion of the bacterial population irregularly scattered on the primary root surface (Fig 4A) and the junction of the primary and lateral roots (Fig 4B). Bacterial cells tended to gathered in the elongation zone of roots (Fig 4C), but fewer gathered at the top of the root tip, being consistent with the results obtained in wheat and maize. At the same time, some bacterial cells entered into the root hair (Fig 4D). Three to five days after inoculation, linear distribution of bacterial cells was observed in the root surface (Fig 4E), being consistent with the results observed in wheat and maize experiments. Longitudinal sections showed that the bacterial cells had invaded inside the root cortex (Fig 4F and 4G). Meanwhile, bacterial cells began to gather and colonize in the region of the lateral root primordium (Fig 4H), suggesting that bacterial cells tended to gather in the region of the actively dividing tissues. The colonization pattern in cucumber was similar to that obtained in maize. The longitudinal and transverse sections revealed that after seven day of inoculation, the bacterial cells invaded into the root endodermis and xylem vessels of the cucumber stems (Fig 4I and 4J). The longitudinal and transverse sections also showed that ten days after inoculation, the bacterial cells were found in leaf vein (Fig 4K) and within the leaves (Fig 4L).

## Discussion

In this study, the GFP-labelled *P*. *polymyxa* WLY78 was used to inoculate wheat, maize and cucumber seedlings grown in the gnotobiotic system. Observation by confocal laser scanning microscope reveals that in maize and cucumber seedlings, bacterial cells were colonized in epidermal and cortical cells, intercellular spaces and vascular system of root, stem and leaf tissue interiors besides on root surfaces. Higher densities of the bacterial cells in roots, stems and leaves indicated that *P*. *polymyxa* WLY78 cells could migrate from roots to stems and leaves of maize and cucumber. However, we found that wheat seedlings grown in both the gnotobiotic system and in soil, the majority of the bacterial cells were distributed on root surface and epidermis, and only a few cells are located within cortical cells in both systems. The results obtained in wheat are a little different from those from maize and cucumber. The result that only a few bacterial cells are located within cortical cells of wheat may be due to the reason that we could not expertly use confocal laser scanning microscope to observe bacterial colonization pattern at the beginning of this study.

Our recent studies have shown that *P*. *polymyxa* WLY78 has the abilities of phosphate solubilization and IAA production besides nitrogen-fixation [6]. We deduce that nitrogen fixation, IAA production and phosphate solubilization performed by *P*. *polymyxa* WLY78 inside roots, stems and leaves and on root surfaces are contributive to plant-growth promotion of the bacterium when plants were inoculated with this bacterium.

The current colonization pattern of the *P*. *polymyxa* WLY78 is similar to those of the Gram-negative diazotroph *Azospirillun brasilense* Yu62 [12, 13] and the Gram-positive *Bacillus megaterium* C4 [14], suggesting that *P*. *polymyxa* WLY78 is an associated nitrogen-fixer. Our data are a little different from Timmusk et al’s report [15] that *P*. *polymyxa* B1 colonized predominantly on the root tip. Our data support the previous results that the colonization pattern is closely related to bacterial strains. This study will provide some information for developing nitrogen-fixing cereals [16].
